# Pharmacokinetics and metabolism of lidocaine HCl 2% with epinephrine in horses following a palmar digital nerve block

**DOI:** 10.1186/s12917-023-03787-x

**Published:** 2023-10-30

**Authors:** Heather K. Knych, Scott Katzman, Daniel S. McKemie, Rick M. Arthur, Jeff Blea

**Affiliations:** 1 K.L. Maddy Equine Analytical Chemistry Laboratory, (Pharmacology Section) School of Veterinary Medicine, University of California, Davis, 620 West Health Science Drive, Davis, CA 95616 USA; 2 Department of Molecular Biosciences, School of Veterinary Medicine, University of California, Davis, CA USA; 3 Department of Surgical and Radiological Sciences, School of Veterinary Medicine, University of California, Davis, CA USA; 4 School of Veterinary Medicine, University of California, Davis, CA USA

**Keywords:** Horse, Palmar digital nerve block, Lidocaine, Epinephrine, Metabolism, Pharmacokinetics, Horseracing

## Abstract

**Background:**

Lidocaine is a local anesthetic that is sometimes administered in combination with epinephrine. The addition of epinephrine increases the time lidocaine remains at the site of administration, thus prolonging the duration of effect. Due to their potential to prevent the visual detection of lameness, the administration of local anesthetics is strictly regulated in performance and racehorses. Recent reports of positive regulatory findings for lidocaine in racehorses suggests a better understanding of the behavior of this drug is warranted. The objective of the current study was to describe serum and urine concentrations and the pharmacokinetics of lidocaine and its primary metabolites following administration in combination with epinephrine, as a palmar digital nerve block in horses. Twelve horses received a single administration of 1 mL of 2% lidocaine HCl (20 mg/horse) with epinephrine 1:100,000, over the palmar digital nerve. Blood samples were collected up to 30 h and urine samples up to 48 h post administration. Lidocaine and metabolite concentrations were determined by liquid chromatography- mass spectrometry and pharmacokinetic (non-compartmental and compartmental) analysis was performed.

**Results:**

Serum concentrations of lidocaine and 3-hydroxylidocaine were above the LOQ of the assay at 30 h post administration and monoethylglycinexylidide (MEGX) and glycinexylidide (GX) were below detectable levels by 24 and 48 h, respectively. In urine, lidocaine, MEGX and GX were all non-detectable by 48 h post administration while 3-hydroxylidocaine was above LOQ at 48 h post administration. The time of maximal concentration for lidocaine was 0.26 h (median) and the terminal half-life was 3.78 h (mean). The rate of absorption (Ka) was 1.92 1/h and the rate of elimination (Kel) was 2.21 1/h.

**Conclusions:**

Compared to previous reports, the terminal half-life and subsequent detection time observed following administration of lidocaine in combination with epinephrine is prolonged. This is likely due to a decrease in systemic uptake of lidocaine because of epinephrine induced vasoconstriction. Results of the current study suggest it is prudent to use an extended withdrawal time when administering local anesthetics in combination with epinephrine to performance horses.

**Supplementary Information:**

The online version contains supplementary material available at 10.1186/s12917-023-03787-x.

## Background

Local anesthetics reduce transmission of electrical impulses in nerve fibers by blocking the influx of sodium ions through voltage gated channels, thus reducing sensation in a localized area. 1 The most common use for local anesthetics in performance horses is for the assessment and localization of lameness as part of a lameness evaluation. Lidocaine is the prototypical amide local anesthetic and is characterized by a rapid onset of effect (2–5 min), an intermediate duration of action and reduced systemic toxicity compared to other local anesthetics. 2 To increase the time lidocaine remains at the site of administration and thus prolong the duration of effect, the drug is often formulated in combination with epinephrine. 3 Epinephrine causes vasoconstriction, thus decreasing the rate of clearance from the target site. 4.

Lidocaine is extensively metabolized in most species including horses, and several different metabolites have been identified. Lidocaine undergoes N- dealkylation, a reaction carried out by CYP3A4 in humans 5, generating the monoethylglycinexylidide (MEGX) metabolite. While the enzyme responsible for this reaction in horses has not been identified, MEGX is a major metabolite in this species. 6, 7 The MEGX metabolite is further converted to glycinexylidide (GX). Other primary metabolites of lidocaine include 3- and 4-hydroxylidocaine. 7, 8.

Due to their potential to prevent the visual detection of lameness, the administration of local anesthetics is strictly regulated in performance and racehorses. The pharmacokinetics of the local anesthetic, lidocaine following intravenous, topical and subcutaneous administration in horses has been reported previously. 6, 7 However, recent reports of positive regulatory findings for lidocaine in racehorses suggests a better understanding of the behavior of this drug is warranted, and specifically, when lidocaine is administered as a nerve block in combination with epinephrine. To that end, the objective of the current study was to describe serum and urine concentrations and the pharmacokinetics of lidocaine and its primary metabolites following administration in combination with epinephrine, as a palmar digital nerve block in horses.

## Results

### Concentration determination and pharmacokinetic analysis

The liquid chromatography-tandem mass spectrometry (LC-MS/MS) instrument response for all analytes was linear and gave correlation coefficients of 0.99 or better. The precision and accuracy of the assay was determined by assaying quality control samples in replicates (n = 6) for all analytes. Accuracy was reported as percent nominal concentration and precision as percent relative standard deviation (Table 1). The technique was optimized to provide a limit of quantitation (LOQ) in serum of 0.005 ng/mL for lidocaine and 3-hydroxylidocaine and 0.05 ng/mL for MEGX and GX. The limit of detection (LOD) in serum was approximately 0.0025 ng/mL for lidocaine and 3-hydroxylidocaine and 0.025 ng/mL for MEGX and GX. For urine, the LOQ was 0.05 ng/mL for lidocaine, 0.1 ng/mL for 3-hydroxylidocaine and MEGX and 0.2 ng/mL for GX. The urine LOD was 0.025 ng/mL for lidocaine, 0.05 ng/mL for 3-hydroxylidocaine and MEGX and 0.1 ng/mL for GX.

**Table 1:** Accuracy and precision values for LC-MS/MS analysis of lidocaine, 3-hydroxylidocaine, monoethylglycinexylidide (MEGX) and glycinexylidide (GX) in equine serum (A) and urine (B)

Analyte	Concentration (ng/mL)	Intra-day accuracy (% nominal concentration)	Intra-day precision (% relative SD)	Inter-day accuracy (% nominal concentration)	Inter-day precision (% relative SD)
**A.)**					
Lidocaine					
	0.15	98.0	1.0	97.0	3.0
	1.0	100	6.0	101	5.0
	5.0	104	2.0	104	3.0
3-hydroxylidocaine					
	0.15	113	10.0	105	6.0
	1.0	115	6.0	106	6.0
	5.0	111	11.0	106	9.0
MEGX					
	0.15	110	4.0	106	5.0
	1.0	113	4.0	107	4.0
	5.0	114	6.0	104	5.0
GX					
	0.15	105	6.0	100	8.0
	1.0	115	8.0	106	8.0
	5.0	106	4.0	105	7.0
**B.)**					
Lidocaine					
	0.6	105	9.0	104	8.0
	5.0	118	7.0	112	5.0
	800	103	3.0	103	4.0
3-hydroxylidocaine					
	0.6	96.0	6.0	103	7.0
	5.0	103	6.0	105	7.0
	800	100	3.0	104	6.0
MEGX					
	0.6	101	2.0	104	3.0
	5.0	103	3.0	102	2.0
	800	102	2.0	102	3.0
GX					
	0.6	90.0	2.0	93.0	3.0
	5.0	104	6.0	101	4.0
	800	103	6.0	101	3.0

**Fig. 1:**
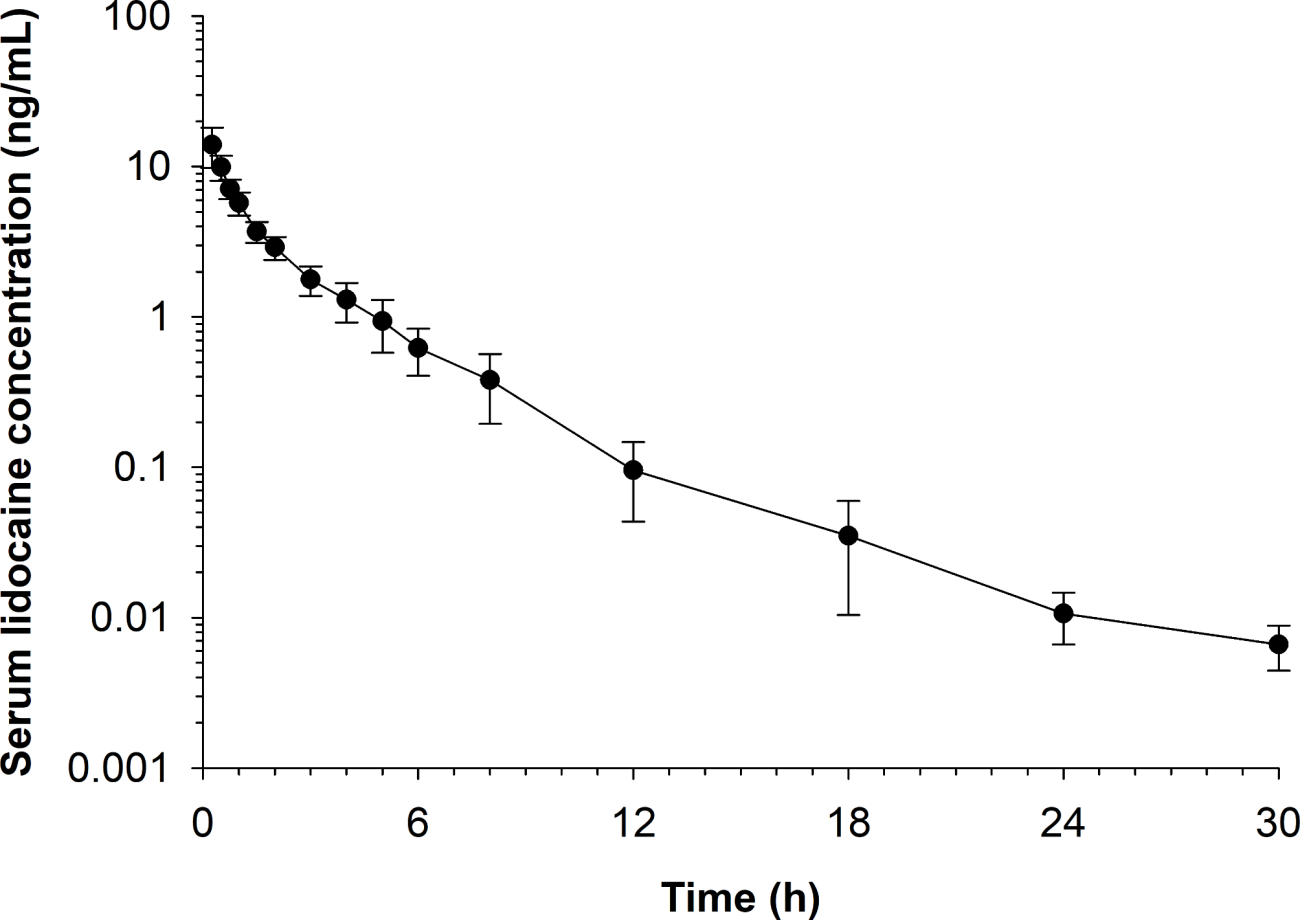
Mean (± SD) serum lidocaine concentrations over time following administration of 1 mL of Lidocaine HCl 2% (20 mg) with Epinephrine (1:100,000) as a palmar digital nerve block in horses (n = 12)

**Fig. 2:**
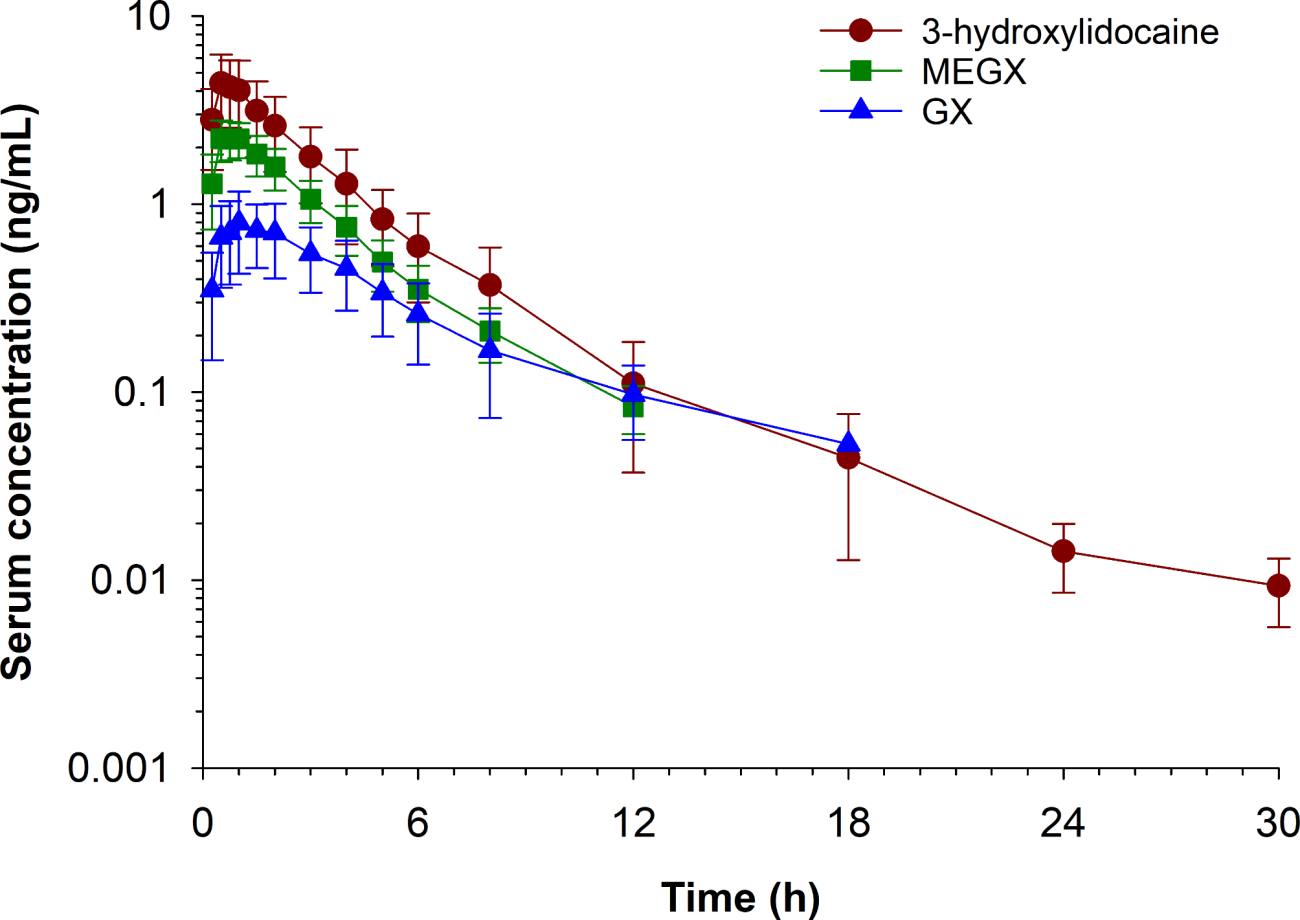
Mean (± SD) serum lidocaine metabolite concentrations over time following administration of 1 mL of Lidocaine HCl 2% (20 mg) with Epinephrine (1:100,000) as a palmar digital nerve block in horses (n = 12)

Serum concentration time curves for lidocaine, 3-hydroxylidocaine, MEGX and GX are depicted in Figs. 1 and 2, respectively. Mean (± SD) serum concentrations of lidocaine and its metabolites are presented in Table 2. Concentrations of lidocaine and 3-hydroxylidocaine were above the LOQ (0.005 ng/mL) of the assay at 30 h post administration (last time point measured). MEGX and GX were below detectable levels by 24 and 48 h, respectively.

**Table 2:** Mean (± SD) serum concentrations of lidocaine, 3-hydroxylidocaine, monoethylglycinexylidide (MEGX) and glycinexylidide (GX) following administration of 1 mL of Lidocaine HCl 2% (20 mg) with Epinephrine (1:100,000) as a palmar digital nerve block to horses (n = 12)

Time (h)	Lidocaine	3-hydroxylidocaine	MEGX	GX
	Concentration (ng/mL)			
0	ND	ND	ND	ND
0.25	9.39 ± 2.70	2.81 ± 1.29	1.29 ± 0.55	0.35 ± 0.20
0.5	6.89 ± 1.19	4.40 ± 1.86	2.23 ± 0.56	0.67 ± 0.31
0.75	4.77 ± 0.63	4.19 ± 1.63	2.22 ± 0.51	0.71 ± 0.33
1.0	4.01 ± 0.68	4.03 ± 1.77	2.23 ± 0.47	0.80 ± 0.37
1.5	2.50 ± 0.30	3.14 ± 1.36	1.85 ± 0.45	0.73 ± 0.27
2.0	2.01 ± 0.37	2.61 ± 1.12	1.58 ± 0.39	0.70 ± 0.30
3.0	1.21 ± 0.26	1.79 ± 0.78	1.06 ± 0.27	0.55 ± 0.21
4.0	0.87 ± 0.28	1.28 ± 0.67	0.75 ± 0.22	0.46 ± 0.18
5.0	0.63 ± 0.25	0.83 ± 0.36	0.49 ± 0.15	0.34 ± 0.14
6.0	0.42 ± 0.17	0.60 ± 0.30	0.35 ± 0.12	0.26 ± 0.12
8.0	0.25 ± 0.13	0.37 ± 0.22	0.21 ± 0.07	0.17 ± 0.09
12.0	0.06 ± 0.04	0.11 ± 0.07	0.08 ± 0.02	0.02 ± 0.01
18.0	0.02 ± 0.02	0.04 ± 0.03	<LOQ	0.10 ± 0.04
24.0	0.01 ± 0.0	0.01 ± 0.01	ND	0.05 ± 0.0
30.0	0.01 ± 0.0	0.01 ± 0.0	ND	ND

ND, not detected; LOQ, limit of quantitation

Pharmacokinetic parameters for lidocaine and metabolites following NCA are listed in Table 3. The time of maximum concentration (T_max_) for lidocaine ranged from 0.25 to 0.5 h and the mean terminal half-life was 3.78 h (mean). For the metabolites, based on mean values, the maximum serum concentration (C_max_) was greatest for 3-hydroxylidocaine followed by MEGX and GX.

**Table 3:** Pharmacokinetic parameters for lidocaine, 3-hydroxylidocaine, monoethylglycinexylidide (MEGX) and glycinexylidide (GX) following administration of 1 mL of Lidocaine HCl 2% (20 mg) with Epinephrine (1:100,000) as a palmar digital nerve block to horses (n = 12). Values are reported as geometric mean and range, unless otherwise indicated

Parameter	Lidocaine	3-hydroxylidocaine	MEGX	GX
C_max_ (ng/mL)	13.4 (7.67–20.1)	4.55 (2.51–7.66)	2.32 (1.40–3.29)	0.750 (0.369–1.52)
T_max_ (h)^*^	0.26 (0.25–0.5)	0.5 (0.5-1.0)	0.75 (0.5-1.0)	1.0 (0.5-2.0)
AUC_inf_ (h*ng/mL)	20.5 (15.7–26.1)	14.5 (6.64-24.0)	7.90 (5.17–10.1)	3.94 (1.52–8.17)
AUC extrap (%)	0.28 (017 − 0.60)	0.41 (0.25–0.60)	3.54 (1.76–9.10)	8.54 (3.24–17.5)
Lambda_z_ (1/h)	0.183 (0.092–0.247)	0.167 (0.121–0.323)	0.305 (0.217–0.590)	0.237 (0.154–0.346)
Half-life (h) ^†^	3.78 (2.80–7.57)	3.97 (2.14–5.75)	2.15 (1.17–3.61)	2.84 (2.01–4.49)

*, median; ^†^, harmonic mean; C_max_ is the maximum measured serum concentration; T_max_ is the time of the maximum serum concentration; AUC_inf_ is the area under the curve extrapolated to infinity; AUC extrap is the percentage of the area under the curve that is extrapolated; Lambda_z_ is the slope of the terminal elimination curve, half-life is the terminal half-life

The final pharmacokinetic model was a 2-compartment model with linear absorption and without a lag time, parameterized with respect to clearance. A multiplicative residual error model was used. Due to poor model fit, it was not possible to incorporate the metabolites into the model. Pharmacokinetic parameters (estimate, standard error (SE) and coefficient of variation) for the model are listed in Table 4. For lidocaine, the rate of absorption (Ka) was 1.92 1/h and the rate of elimination (Kel) was 2.21 1/h.

**Table 4:** Estimated pharmacokinetic parameters of lidocaine, 3-hydroxylidocaine, monoethylglycine xylidide (MEGX) and glycinexylidide (GX) following administration of 1 mL of Lidocaine HCl 2% (20 mg) with Epinephrine (1:100,000) as a palmar digital nerve block to horses (n = 12). Parameters were generated using non-liner mixed-effect modeling

Parameter	Estimate	SE	CV (%)
tvKa (1/h)	1.92	0.43	22.6
tvV (L)	410.4	106.8	26.0
tvV2 (L)	1579.7	201.8	12.8
tvCl (mL/min)	15,090.8	2057.8	13.6
tvCl2 (mL/min)	8452.6	1805.2	21.4
stdev0	0.378	0.071	18.8
Ke (1/h)	2.21	0.758	34.4
Alpha (1/h)	3.56	1.27	35.7
Beta (1/h)	0.199	0.014	6.95
AUC (ng*h*mL)	22.1	3.01	13.6
Alpha HL (h)	0.194	0.069	35.7
Beta HL (h)	3.49	0.242	6.95
Ke HL (h)	0.314	0.108	34.4
Ka HL (h)	0.361	0.082	22.6

tv represents typical values; tvKa, rate of absorption; tvV, the value of the central compartment; tvV2, the value of the peripheral compartment; tvCl, the clearance of drug from plasma; tvCl2, the clearance of drug from the peripheral compartment; stdev0 the estimated residual standard deviation for plasma data; Ke, elimination rate constant; Alpha and beta, slopes for the modeled equation; AUC, area under the concentration time curve; Alpha HL, phase 1 half-life; Beta HL, phase 2 half-life; Ke HL, elimination half-life, Ka HL, absorption half-life

Urine concentrations of lidocaine and metabolites are listed in Table 5. Lidocaine, MEGX and GX were all non-detectable by 48 h post administration. The concentration of 3-hydroxylidocaine was 1.24 ± 0.04 ng/mL (mean ± SD) at 48 h post administration.

**Table 5:** Urine concentrations of lidocaine, 3-hydroxylidocaine, monoethylglycinexylidide (MEGX) and glycine xylidide (GX) following administration of 1 mL of Lidocaine HCl 2% (20 mg) with Epinephrine 1:100,000 to 12 horses

Time (h)	Lidocaine	3-hydroxylidocaine	MEGX	GX
	Concentration (ng/mL)			
4.0	10.7 ± 5.25	681.8 ± 302.0	16.6 ± 6.95	87.1 ± 38.1
24.0	0.09 ± 0.04	7.09 ± 5.65	0.58 ± 0.37	0.77 ± 0.85
48.0	ND	1.24 ± 0.04	ND	ND

ND, not detected

## Discussion

The current study reports blood and urine concentrations and the pharmacokinetics of lidocaine and its metabolites following administration of the drug in combination with epinephrine, as a palmar digital nerve block to horses. The pharmacokinetics of lidocaine following subcutaneous administration have been reported previously in horses, 6, 7 however, there are no published reports describing lidocaine pharmacokinetics when administered in combination with epinephrine, in this species.

In the current study, a two-compartment model without a lag time best fit lidocaine concentration data following subcutaneous administration. This agrees with previous reports describing the pharmacokinetics of lidocaine HCl in horses. 6, 9 In the present study, the mean terminal half-life of lidocaine (3.78 h), determined using NCA, was longer than that reported by Soma and colleagues (0.18 h). 6 However, it should be noted that the analytical method utilized in the current study was more sensitive as compared to the previous study. 6 This has likely allowed for more complete characterization of the terminal portion of the concentration time curve, making the most likely explanation for the discrepancy between the two studies the use of different drug formulations. As described previously, the disposition of lidocaine in combination with epinephrine prolongs the terminal half-life of lidocaine. 10 Vasoconstriction, stimulated by the epinephrine in the administered formulation can lead to a decrease in the rate of uptake (absorption) into the systemic circulation, making the rate of absorption the determining factor for the persistence of lidocaine. 10 The prolonged terminal half-life reported here is likely a result of prolonged systemic absorption as opposed to delayed elimination. Notably, lidocaine was not administered intravenously in the current study, making it impossible to confirm this hypothesis. However, a previous study reports an elimination half-life of 0.17 h following intravenous administration of lidocaine, 6 suggesting that the prolonged residence time of lidocaine in the current study is largely influenced by rate of absorption. The relatively slower rate of absorption (Ka: 1.92 1/h), compared to the rate of elimination (Kel: 2.21 1/h), further supports this theory.

A notable limitation in the current study was the minimal number of samples available to characterize the absorption phase. The first sample was not collected until 15 min post administration, with the mean lidocaine T_max_ occurring at 15 min. Although the %CV for this parameter in the fitted model was deemed acceptable (< 25%), earlier time points may have allowed for more complete characterization of the absorption phase.

Although the most likely explanation for the differences in pharmacokinetic values between the Soma et al. study 6 and the current report is the difference in drug formulations, it is also notable that the injection site was different in the two studies. Soma and colleagues administered lidocaine over the carpus, 6 while in the current study, drug was administered in the distal extremity. As has been reported by other investigators, the vascularity of the injection site can alter the rate of uptake into the systemic circulation, following subcutaneous administration. 11.

In agreement with previous studies, the primary metabolites identified in the current study were 3-hydroxylidocaine, MEGX and GX. 6, 12 Serum concentrations of lidocaine and 3-hydroxylidocaine were above the LOQ (0.005 ng/mL) at 30 h post administration (the last time point sampled) and the terminal half-life in blood was comparable for lidocaine and 3-hydroxylidocaine. Attempts at incorporating the metabolites into the parent NLME model and subsequent generation of a combined parent-metabolite pharmacokinetic model were unsuccessful. Additional information that would have likely enhanced the ability to generate a combined model include the volume of distribution and/or clearance of the metabolites following intravenous administration and/or the determination of urine clearance. 13 Clearance and volume of distribution of the lidocaine metabolites in horses has not been reported. Although these pharmacokinetic parameters have been reported for MEGX in humans, 14 and since metabolism can vary between species, utilizing these values in the current model was not appropriate. With respect to urine clearance, while concentrations were determined, volumes were not measured, making calculation of urinary clearance of the metabolites not possible.

## Conclusions

Precautions, specifically adherence to recommended withdrawal times, should be taken when administering local anesthetics such as lidocaine to performance horses. Compared to previous reports, the difference in pharmacokinetics reported here, specifically the terminal half-life and subsequent detection time observed following administration of 2% lidocaine in combination with epinephrine (1:100,000), also underscores the importance of being cognizant of the specific formulation. Results of the current study suggest it is prudent to use an extended withdrawal time when administering local anesthetics in combination with epinephrine to performance horses.

## Methods

### Animals

Twelve healthy, university-owned, treadmill-exercised Thoroughbred research horses (5 mares and 7 geldings; 4–7 years; weight: 457–576 kg) were included in the study. No medications for were administered for a minimum of two weeks prior to drug administration. Prior to inclusion, a physical examination, complete blood count (CBC) and a serum biochemistry panel were performed for each horse. The CBC and biochemistry panel were performed by the Clinical Pathology Laboratory of the William R. Pritchard Veterinary Medical Teaching Hospital of the University of California, Davis. The study was approved by the University of California at Davis’ Institutional Animal Care and Use Committee of the (IACUC #22,110).

### Instrumentation and drug administration

For sample collection, a 14- gauge intravenous catheter was placed in one external jugular vein, using aseptic technique, prior to administration of drug. Horses received a single subcutaneous injection of 1 mL of 2% lidocaine HCl (20 mg/horse) with Epinephrine 1:100,000 (Med-vet International, Mettawa, IL) over the palmar digital nerve. The palmar digital neurovascular bundle was palpated immediately proximal to the ungular cartilages of the distal phalanx. The area was subsequently prepared for injection using gauze swab saturated with 70% isopropyl alcohol. A 25-gauge hypodermic needle was placed percutaneously in a proximal to distal direction axial to the neurovascular bundle, immediately adjacent to the palmar digital nerve. The syringe containing the lidocaine and epinephrine was then attached to the hub of the needle and the drug combination deposited subcutaneously.

### Sample collection

Blood samples were collected at time 0 (prior to drug administration) and at 15, 30, and 45 min, and 1, 1.5, 2, 3, 4, 5, 6, 8, 12, 18, 24 and 30 h post administration for determination of lidocaine concentration. Samples were collected into blood tubes devoid of anti-coagulant (red top) and were allowed to sit at room temperature for approximately 20 min, before centrifugation at 3000 x g. Serum was immediately transferred to storage cryovials and stored at -20^◦^ C (4 weeks) until analysis for determination of lidocaine concentrations.

Urine samples were collected at 4, 24 and 48 h post drug administration by free catch. Samples were stored at -20^◦^ C (4 weeks) until analyzed for determination of lidocaine concentrations.

### Determination of drug and metabolite concentrations

#### Serum

Lidocaine (Cerilliant, Round Rock, TX), 3-hydroxylidocaine (Frontier BioPharm; Richmond, KY), GX (Toronto Research Chemicals, Toronto, ON) and MEGX (Cerilliant, Round Rock, TX) were combined into one working solution. Serum calibrators (0.005 to 20 ng/mL) were prepared by dilution of the working standard solutions with drug free equine serum. Negative control and calibration curve samples were prepared fresh for each quantitative assay. Quality control samples (drug free equine serum containing analytes at three concentrations within the standard curve) were included with each sample set as an additional check of accuracy.

Prior to analysis, 0.25 mL of serum was diluted with 0.1 mL of water containing 25 ng/mL of d10-lidocaine internal standard (Toronto Research Chemicals, Toronto, ON) and 0.2 mL of β-glucuronidase enzyme, (Sigma Aldrich, St Louis, MO) at 10,000 Units/mL in pH 5, 1.6 M acetate buffer. The pH of the samples was adjusted to 5.0 ± 0.5 with 2 N NaOH or 2 N HCl, as necessary, and heated in a water bath at 37 °C for 2 h. After cooling to room temperature, 0.2 mL of 0.5 N NaOH was added to adjust the pH to 10.0 ± 0.5 with. Methyl tert-butyl ether (MTBE; 3mL) was added to each serum sample, and the samples were mixed by rotation for 20 min at 40 revolutions per minute. Samples were then centrifuged at 3300 rpm (2260 g) for 5 min at 4 °C. The top organic layer transferred to glass tubes and samples were dried under nitrogen and dissolved in 120 uL of 5% acetonitrile in water with 0.2% formic acid. The sample (30 uL ) was injected into the LC-MS/MS system.

Liquid chromatography-tandem mass spectrometry using positive heated electrospray ionization (HESI(+)) was used to measure lidocaine and metabolite concentrations. A TSQ Altis triple quadrupole mass spectrometer coupled with a Vanquish liquid chromatography system (Thermo Scientific, San Jose, CA) was used for quantitative analysis. The spray voltage was 3500 V, the vaporizer temperature 350ºC, and the sheath and auxiliary gas were 50 and 10 respectively (arbitrary units). To optimize product masses and collision energies of each analyte standards were infused into the TSQ Altis. An ACE 3 C18 10 cm x 2.1 mm 3 μm column (Mac-Mod Analytical, Chadds Ford, PA) and a linear gradient of ACN in water with a constant 0.2% formic acid at a flow rate of 0.35 ml/min was used for chromatography. Initially the ACN concentration was held at 3% for 0.5 min, then ramped to 90% over 6.0 min and held at that concentration for 0.2 min, before re-equilibrating at initial conditions for 4.6 min.

Selective reaction monitoring (SRM) of initial precursor ion for lidocaine (mass to charge ratio 235.2 *(m/z)*), 3-hydroxylidocaine (mass to charge ratio 251.1 *(m/z)*), GX (178.9 *(m/z)*), MEGX (207 *(m/z)*), and the internal standard d10-lidocaine (245.2 *(m/z)*). The response for the product ions for lidocaine (*m/z* 30.2, 58.1, 86.1), 3-hydroxylidocaine (*m/z* 30.3, 58.1, 86.1), GX (*m/z* 122), MEGX (*m/z* 58) and the internal standard d10-lidocaine (*m/z* 64.1, 96.2) were plotted and peaks at the proper retention time integrated using Quanbrowser software (Thermo Scientific). Generation of calibration curves and quantitation of analytes by linear regression analysis was conducted using Quanbrowser software. For all calibration curve, a weighting factor of 1/X was used.

#### Urine

Working solutions for urine analysis were the same as described above for serum. Calibrators (0.05 to 1,500 ng/mL) were prepared by dilution of the working standard solutions with drug free equine urine. Urine calibration curves and negative control samples were prepared fresh for each quantitative assay. As an additional check of accuracy, quality control samples were included with each sample. The extraction method for the urine was the same as the serum except the sample size for urine was 0.5 mL, samples were hydrolyzed at 65 °C with 99 min of sonication, and after hydrolysis the urine was adjusted to pH 9. Methyl tert-butyl ether (5 mL) was used for extraction, samples were redissolved in 150 uL of 5% acetonitrile in water, with 0.2% formic acid and 20 uL into the LC-MS/MS system. Detection and quantification in urine was the same as for the serum.

### Pharmacokinetic analysis

Pharmacokinetic analyses of lidocaine and metabolite serum concentration data were conducted using non-compartmental analysis (NCA) and a commercially available pharmacokinetic software program (Phoenix Winnonlin v8.3, Certara, Princeton, NJ). Maximum concentrations (C_max_) and the time of maximum concentration (T_max_) were determined directly from the concentration data.

After performing NCA, pharmacokinetic modeling using a nonlinear mixed effect modeling (NLME) approach with the Phoenix NLME software program and concentration data was conducted. Two and three-compartment models with saturable and linear absorption, with and without a lag time and with different error models were assessed using lidocaine concentration data. Following selection of the best fit model for lidocaine, the metabolites were added to attempt to generate a parent-metabolite pharmacokinetic model. The goodness of fit of the models was determined by visual analysis of observed compared with predicted concentration graphs and residual plots, as well as CV, Akaike Information Criterion, and % CV of parameter estimates.

### Electronic supplementary material

Below is the link to the electronic supplementary material.

Supplementary Material 1

## Data Availability

The datasets used and/or analyzed during the current study are available from the corresponding author on reasonable request.

## Abbreviations

AUC: Area under the curve
C_max_: Maximum concentration
CBC: Complete blood count
CYP3A4: Cytochrome P450 3A4
GX: Glycinexylidide
Ka: Rate of absorption
Kel: Rate of elimination
LC-MS/MS: Liquid chromatography mass spectrometry/mass spectrometry
LOQ: Limit of quantitation
MEGX: Monoethylglycinexylidide
NCA: Non-compartmental analysis
NLME: Non-linear mixed effects model
SRM: Selective reaction monitoring
T_max_: time of maximum concentration

## References

[1] Becker DE, Reed KL (2012). Local anesthetics: review of pharmacological considerations. Anesth Prog.

[2] Vickroy TW. Local anesthetics. In J.E. Riviere & M.G. Papich 9Eds.), Veterinary Pharmacology & Therapeutics. 10th ed. Wiley Blackwell; 2018.

[3] Alvarez AV, Schumacher J, DeGraves FJ (2018). Effect of the addition of epinephrine to a lidocaine solution on the efficacy and duration of palmar digital nerve blocks in horses with naturally occurring forefoot lameness. Am J Vet Res.

[4] Bernards CM, Kopacz DJ (1999). Effect ofeEpinephrine on lidocaine clearance in vivo: a microdialysis study in humans. Anesthesiology.

[5] Singer MI, Shapiro LE, Shear NH, Cytochrome (1997). P-450 3A: interactions with dermatologic therapies. J Am Acad Dermatol.

[6] Soma LR, You Y, Robinson MA, Boston RC (2018). Pharmacokinetics of intravenous, subcutaneous, and topical administration of lidocaine hydrochloride and metabolites 3-hydroxylidocaine, monoethylglycinexylidide, and 4-hydroxylidocaine in horse. J Vet Pharmacol Ther.

[7] Harkins JD, Mundy GD, Woods WE (1998). Lidocaine in the horse: its pharmacological effects and their relationship to analytical findings. J Vet Pharmacol Ther.

[8] Soma LR, Behrend E, Rudy J, Sweeney RW (1988). Disposition and excretion of flunixin meglumine in horses. Am J Vet Res.

[9] Minuto J, Bedenice D, Ceresia M, Zaghloul I, Böhlke M, Mazan MR (2022). Clinical effects and pharmacokinetics of nebulized lidocaine in healthy horses. Front Vet Sci.

[10] Moore PA, Hersh EV, Papas AS (2008). Pharmacokinetics of lidocaine with epinephrine following local anesthesia reversal with phentolamine mesylate. Anesth Prog.

[11] Scott DB, Jebson PJ, Braid DP, Ortengren B, Frisch P (1972). Factors affecting plasma levels of lignocaine and prilocaine. Br J Anaesth.

[12] Dickey EJ, McKenzie HC, Brown KA, de Solis CN (2008). Serum concentrations of lidocaine and its metabolites after prolonged infusion in healthy horses. Equine Vet J.

[13] Ahlers SJGM, Välitalo PAJ, Peeters MYM (2015). Morphine glucuronidation and elimination in intensive care patients: a comparison with healthy volunteers. Anesth Analg.

[14] Thomson AH, Elliott HL, Kelman AW, Meredith PA, Whiting B (1987). The pharmacokinetics and pharmacodynamics of lignocaine and MEGX in healthy subjects. J Pharmacokinet Biopharm.

