# Supplementary figures and images for: Pharmacokinetics and metabolism of lidocaine HCl 2% with epinephrine in horses following a palmar digital nerve block
Source: BMC Vet Res. 2023 Oct 30;19:225. doi: 10.1186/s12917-023-03787-x (PMC10614310; doi:10.1186/s12917-023-03787-x)

A.)

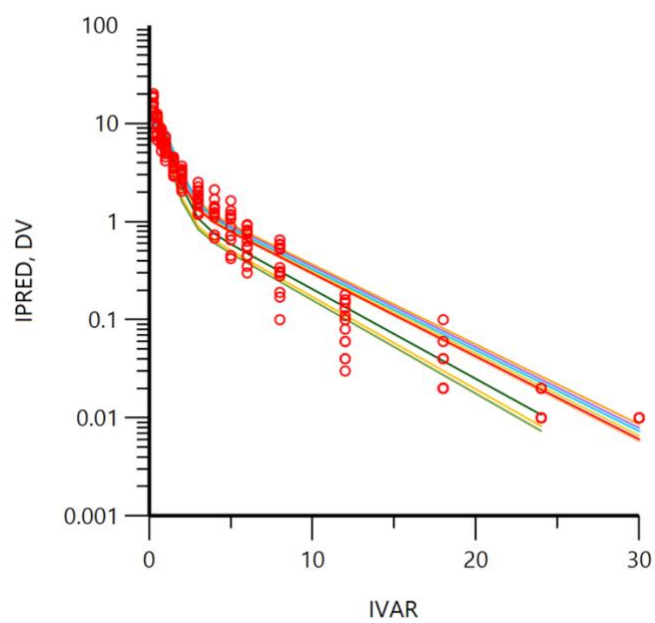

B.)

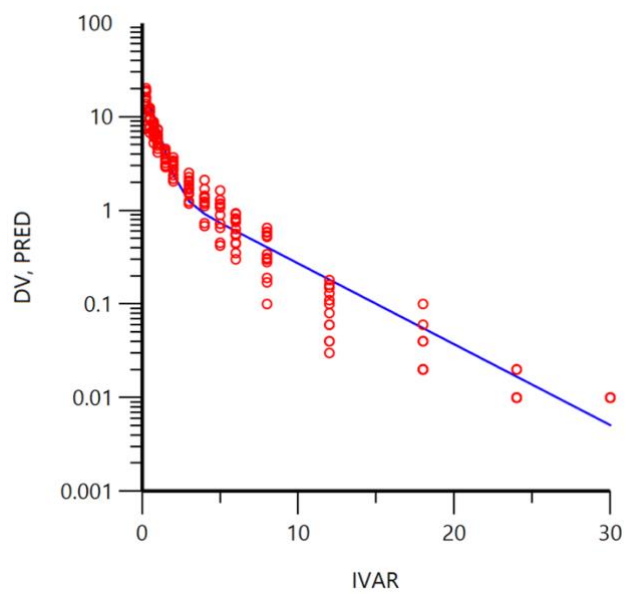

A.)

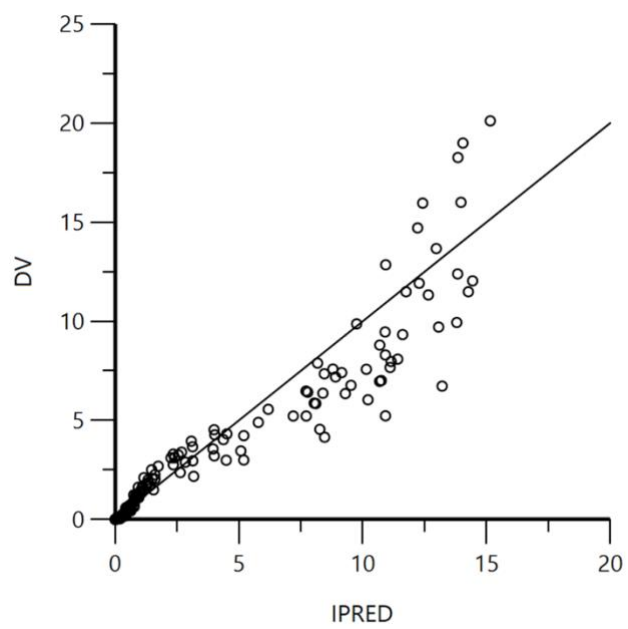

B.)

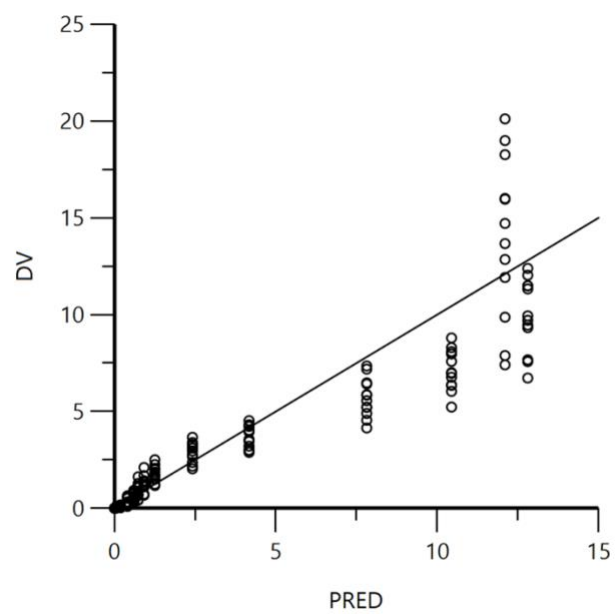

Supplement: Supplementary file 1 — Supplementary Material 1 [file 12917_2023_3787_MOESM1_ESM.pdf]
